# Ultraprocessed Food Consumption and Obesity Development in Canadian Children

**DOI:** 10.1001/jamanetworkopen.2024.57341

**Published:** 2025-01-31

**Authors:** Zheng Hao Chen, Sara Mousavi, Piushkumar J. Mandhane, Elinor Simons, Stuart E. Turvey, Theo J. Moraes, Padmaja Subbarao, Kozeta Miliku

**Affiliations:** 1 Department of Nutritional Sciences, University of Toronto, Toronto, Ontario, Canada; 2 Department of Pediatrics, University of Alberta, Edmonton, Canada; 3 Faculty of Medicine and Health Sciences, UCSI University, Malaysia; 4 Department of Pediatrics and Child Health, University of Manitoba, Winnipeg, Canada; 5 Department of Pediatrics, BC Children’s Hospital, University of British Columbia, Vancouver, Canada; 6 Department of Pediatrics, Hospital for Sick Children, University of Toronto, Toronto, Ontario, Canada; 7 Department of Physiology, University of Toronto, Toronto, Ontario, Canada; 8 Department of Medicine, McMaster University, Hamilton, Ontario, Canada

## Abstract

**Importance:**

Ultraprocessed foods (UPF), characterized as shelf-stable but nutritionally imbalanced foods, pose a public health crisis worldwide. In adults, UPF consumption is associated with increased obesity risk, but findings among children are inconsistent.

**Objectives:**

To examine the associations among UPF intake, anthropometric adiposity indicators, and obesity status in Canadian children.

**Design, Setting, and Participants:**

In the CHILD Cohort Study, one of the largest prospective, multicenter, population-based pregnancy cohorts in Canada, diet was assessed during the 3-year visit (September 2011 to June 2016), and anthropometric measurements were assessed at the 5-year visit (December 2013 to April 2018). Data analysis was performed between July 1, 2023, and June 30, 2024.

**Exposure:**

Diet intake was assessed using a semiquantitative food frequency questionnaire at 3 years of age. UPFs were identified using the NOVA classification system.

**Main Outcomes and Measures:**

Anthropometric adiposity indicators were measured at 5 years of age and used to calculate age- and sex-standardized *z* scores for body mass index (BMI), waist to height ratio, and subscapular and triceps skinfold thicknesses, and obesity, which was defined using BMI *z* score cutoffs. Multivariable-adjusted regression analyses were used to examine the associations of UPF with adiposity and obesity development, accounting for parental, birth, and early-childhood factors.

**Results:**

Among 2217 participants included in this study, median age at the outcome assessment was 5.0 (IQR, 5.0-5.1) years, and 1175 (53.0%) were males. At 3 years of age, UPF contributed 45.0% of total daily energy intake. UPF energy contribution was higher in males vs females (46.0% vs 43.9%; *P* < .001). Among all participants, higher UPF intake at 3 years of age was associated with higher anthropometric adiposity indicators at 5 years of age, primarily driven by males. In males, every 10% UPF energy increase was associated with higher adiposity indicator *z* scores for BMI (β, 0.08; 95% CI, 0.03-0.14), waist to height ratio (β, 0.07; 95% CI, 0.01-0.12), and subscapular (β, 0.12; 95% CI, 0.06-0.18) and triceps (β, 0.09; 95% CI, 0.03-0.15) skinfold thickness and higher odds of living with overweight or obesity (odds ratio, 1.19; 95% CI, 1.03-1.36). No significant associations were observed among females.

**Conclusions and Relevance:**

In this cohort study of Canadian children, high UPF consumption during early childhood was associated with obesity development, primarily in males. These findings can inform targeted public health initiatives for early childhood centers and caregiver education programs to reduce UPF intake and prevent obesity.

## Key Points

**Question:**

Is ultraprocessed food (UPF) consumption associated with anthropometric adiposity indicators and obesity development among Canadian children?

**Findings:**

In this cohort study of 2217 children, UPF contributed to almost half of their daily energy intake. Higher UPF intake was positively associated with body mass index, waist to height ratio, subscapular and triceps skinfold thickness, and higher odds of living with overweight or obesity.

**Meaning:**

These findings can inform public health messages directed to educate caregivers on the long-term health impact of UPF in relation to the prevention of obesity and obesity-related comorbidities.

## Teaser

This cohort study examines the associations of ultraprocessed food intake with anthropometric adiposity indicators, including body mass index, waist to height ratio, and subscapular and triceps skinfold thicknesses, and obesity status among Canadian children.

## Introduction

Consumption of ultraprocessed food (UPF) is increasing worldwide, with North America in the lead.^1,2^ Canada ranks among the top 5 countries for the highest UPF sales.^2^ UPF are accessible, ready-to-consume, and convenient food options for the consumers.^3^ The processing of UPF and use of emulsifiers and additives often lead to shelf-stable foods, most of which are energy-dense and nutritionally imbalanced (eg, with high levels of added sugars, saturated fats, and sodium).^3^ Over the years, UPF has become widely available in Canadian retail stores,^4^ leading to an increase in consumption.^5^ Data from the 2015 Canadian Community Health Survey found that UPF contributed to more than 50% of daily energy intake for children and adolescents.^6^ Diet is a key modifiable risk factor for many chronic diseases^7^; thus, the increased intake of UPF poses a public health concern.^6^

Concurrent with the rise of UPF consumption, obesity rates are increasing. In 2018, 7.3 million Canadian adults (26.8%) were living with obesity, which rose to 8.3 million (28.2%) 2 years later.^8^ More concerning is that one-third of Canadian children and youths were living with overweight or obesity in 2023.^9^ It is known that childhood obesity develops differently in males and females, with a higher tendency in males.^10^ Given that obesity tracks throughout life,^11^ understanding obesity development in early life is crucial for designing interventions to prevent and reduce its burden.

Adult studies have already demonstrated that high UPF intake is associated with obesity and adiposity-related outcomes.^12,13^ However, associations between UPF intake and obesity and adiposity among children are inconsistent.^14^ Currently, most longitudinal UPF studies in children are conducted in Latin American^15,16,17^ and European^18,19^ cohorts, where UPF consumption tends to be lower than in North America. Some studies suggest that childhood UPF consumption is associated with larger waist circumference^15^ and higher body mass index (BMI),^16,18^ while others found no associations with obesity.^17^ To the best of our knowledge, only 1 cross-sectional Canadian study has examined UPF intake among children aged 1.5 to 5 years and their BMI *z* scores, suggesting no significant associations.^20^ Additionally, it is unknown whether the association between UPF and childhood obesity is sex driven. Dietary habits in early life can track into adulthood,^21^ making the preschool period (in Canada determined as before 4 years of age^22^) an important window for establishing dietary patterns. Understanding the impact of UPF intake during critical windows of development is essential for informing public health messages aiming to reduce obesity burden. Therefore, in the prospective CHILD Cohort Study, we examined the associations of UPF intake with anthropometric adiposity indicators (BMI, waist to height ratio, and subscapular and triceps skinfold thicknesses) and obesity status among Canadian children.

## Methods

### Study Population

The CHILD Cohort Study is a longitudinal, population-based pregnancy cohort study with 4 sites across Canada: Vancouver, Edmonton, Manitoba, and Toronto. Pregnant women were recruited between June 2009 and April 2012 (N = 3621), and remained eligible if they delivered a healthy infant born older than 34 weeks and 4 days of gestation (n = 3454).^23^ For the present study, 3232 children participated in the 3-year visit (September 2011 to June 2016), of whom 2411 had dietary data. Among them, 2217 had measurements of BMI, waist to height ratio, or subscapular and triceps skinfold thicknesses at 5 years of age (December 2013 to April 2018) (eFigure 1 in Supplement 1). This study followed the Strengthening the Reporting of Observational Studies in Epidemiology (STROBE) reporting guidelines for cohorts. Caregivers provided written informed consent. Study ethics were approved by the Human Research Ethics Boards at McMaster University, University of Manitoba, University of Alberta, University of British Columbia, and the Hospital for Sick Children.

### Diet Assessment and UPF Intake

Dietary intake data were collected using a 112-item semiquantitative food frequency questionnaire (FFQ), completed by the caregiver at the 3-year visit. The FFQ was validated in a subset of the FAMILY (Family Atherosclerosis Monitoring in Early Life) study.^24,25^ Each item had a 9-level scale to assess intake frequency in the past month ranging from none to greater than 3 times per day. Total energy intake was calculated from the 2019-2020 Food and Nutrients Database for Dietary Studies from the US Department of Agriculture.^26^

We used the NOVA classification system to define UPF based on the extent and degree of food processing.^27^ Two researchers (Z.H.C. and S.M.) independently mapped all FFQ items into the 4 NOVA groups: (1) unprocessed and minimally processed foods, (2) processed culinary ingredients, (3) processed foods, and (4) UPFs.^27^ Ambiguous items were mapped by consensus, with a conservative approach taken to classifying foods in the lesser processed group when applicable. The percentage of daily energy contributed from UPF intake was calculated by dividing the energy intake from UPF by the total daily energy intake of the whole diet, then multiplied by 100.

### Anthropometric Adiposity Measurements

At the 5-year visits, height was measured in a standing position to the nearest millimeter with a stadiometer (Harpenden; GPM & Holtain), and weight was measured using a calibrated scale. Waist circumference was measured using a nonstretchable measuring tape (Ohaus) with an attached spring scale tension gauge. Subscapular and triceps skinfold thicknesses were measured using skin fold calipers (Harpenden). Standardized age- and sex-adjusted *z* scores were calculated for BMI,^28^ waist to height ratio,^29^ and skinfold thicknesses.^30^ We classified children living with overweight or obesity (BMI *z* score >1) and obesity (BMI *z* score >2) using the World Health Organization cutoffs.^28^

### Covariates

Maternal UPF intake during pregnancy, postsecondary degree educational level, and annual household income were self-reported using questionnaires.^23^ Birth weight and child sex at birth were obtained from medical records. Child ethnicity (questionnaire choices were “Caucasian White” [hereafter, White] and Black, East Asian, First Nations, Hispanic, Middle Eastern, mixed, other, South Asian, and South-East Asian [grouped as other in this analysis]),^31^ breastfeeding exclusivity, having older siblings, study site, season of dietary assessment, and organized physical activity were also obtained via questionnaires.^23^

### Statistical Analysis

Descriptive characteristics were reported as mean (SD) for continuous normally distributed variables, median (IQR) for continuous nonnormally distributed variables, and number (percentage) for categorical variables. We performed nonresponse analyses to compare characteristics of (1) participants included in our study with dietary exposures at 3 years of age and anthropometric data at 5 years of age (n = 2217) and participants enrolled in the CHILD study who did not have any dietary information and outcomes assessed (n = 1237), and (2) participants included in this study (n = 2217) with participants with dietary data at 3 years of age but no outcome data at 5 years of age (n = 194). For these analyses, we used an unpaired *t* test for normally distributed variables, Mann-Whitney test for nonnormally distributed variables, and χ^2^ test for categorical variables. Univariate and multivariable regression analyses were used to explore the association between UPF intake at 3 years of age and anthropometric adiposity indicators and overweight or obesity status at 5 years of age. Two models were considered in our analyses: a basic model (adjusted for total energy intake in kilocalories per day at dietary assessment), and a multivariable-adjusted model (adjusted for maternal postsecondary degree, maternal UPF intake during pregnancy, annual household income, child ethnicity, birth weight, exclusive breastfeeding at 6 months, total energy intake, older siblings, organized physical activity, study site, and season of dietary assessment). Collinearity diagnostic tests were assessed to prevent overadjustment. To assess whether associations differed by child sex, we evaluated the statistical interaction by including the product term with UPF in the model and found significant interaction terms (*P* < .20)^32^ (*P* = .12 for BMI, *P* = .09 for waist to height ratio, *P* = .002 for subscapular skinfold, and *P* = .03 for triceps skinfold). Therefore, in addition to examining the associations among all participants, we also stratified the analyses by males and females. To account for reverse causality, we restricted our analyses to a subset of children with BMI data at 3 years of age, where we additionally accounted for baseline BMI in the final model. We also examined the associations separately among children with normal weight and those with overweight and obesity at 3 years of age.

To evaluate the effectiveness of our UPF mappings, we derived the highly processed foods variable, as defined by Poti et al^33^ at the University of North Carolina (UNC). We reran the analyses using this system as it is more reflective of the North American diet.^33^ In another sensitivity analyses, we accounted for nutrients of concern related to UPF: sugar, sodium, and saturated fat. Furthermore, among 1962 participants with UPF data at 3 and 5 years of age, we accounted for the change in UPF intake. We also assessed UPF intake in units per 100 g, a common alternative measure to account for non–energy-contributing items.^19^ To limit potential bias associated with missing data (ranging from 0.4% to 13.7%), missing values of the covariates were imputed (10 imputations) according to the fully conditional specification method (predictive mean matching), assuming no monotone missing pattern.^34^ We reported the pooled effect estimates after multiple imputations.^35^ Participant characteristics before and after imputations are shown in eTable 1 in Supplement 1. To further assess the risk of potential bias due to missing covariate data, we conducted a complete case analysis among participants with data in all covariates (n = 1617) (eFigure 2 in Supplement 1). Last, we reran the analyses across all 4 study sites.

All statistical analyses were performed on SPSS, version 29.0 (IBM Corporation). Figures were generated using RStudio, version 2024.04.2 (Posit PBC). All statistical analyses were 2 sided with a significance level of α = .05. Data analysis was performed between July 1, 2023, and June 30, 2024.

## Results

### Participant Characteristics

The Table shows the characteristics of the 2217 participants included in this study. The median age at dietary assessment was 3.0 (IQR, 3.0-3.1) years; at outcome assessment, 5.0 (IQR, 5.0-5.1) years (Table). About half of the children were male (1175 [53.0%]) and half female (1042 [47.0%]). In terms of ethnicity, 1447 children were White (65.3%) and 770 (34.7%) were other ethnicities. At 5 years of age, almost 20% of the children had overweight or obesity (257 [11.6%] males and 181 [8.2%] females). Participants who enrolled in the study but did not participate in the follow-up visits (n = 1237) shared similar characteristics to those included in these study analyses (n = 2217) (eg, family income, birth weight, and sex at birth); however, we observed significant statistical differences in maternal postsecondary educational level (679 of 965 [70.4%] vs 1714 of 2167 [79.1%], respectively; *P* < .001) (eTable 2 in Supplement 1). Similarly, there were no statistical differences in birth characteristics, total energy intake, or rates of overweight or obesity between the participants included in our study compared with those who were lost to follow-up at the 5-year visit (n = 194) (eTable 2 in Supplement 1).

**Table.** Characteristics of 2217 Participants Included in the Analysis

Characteristic	Participants
Family^a^	
Maternal postsecondary educational level, No. (%)	1742 (78.6)
Maternal energy intake contributed from UPF, mean (SD), %	46.5 (10.5)
Annual family income, No. (%)	
<$100 000	892 (40.2)
≥$100 000	1178 (53.1)
Prefer not to say	147 (6.6)
Other siblings, No. (%)	1014 (45.7)
Study site, No. (%)	
Edmonton	492 (22.2)
Manitoba	705 (31.8)
Toronto	483 (21.8)
Vancouver	537 (24.2)
Birth^a^	
Child sex, No. (%)	
Male	1175 (53.0)
Female	1042 (47.0)
Birth weight, mean (SD), kg	3.5 (0.5)
Child ethnicity, No. (%)^b^	
White	1447 (65.3)
Others	770 (34.7)
Exclusive breastfeeding at 6 mo, No. (%)	414 (18.7)
Childhood^a^	
Age at dietary assessment, median (IQR), y	3.0 (3.0 to 3.1)
Season of dietary assessment, No. (%)	
Spring	603 (27.2)
Summer	563 (25.4)
Autumn	519 (23.4)
Winter	532 (24.0)
Daily caloric intake, median (IQR), kcal/d	1517.8 (1239.2 to 1858.1)
Daily sodium intake, median (IQR), mg/d	2168.3 (1730.0 to 2780.5)
Daily sugar intake, median (IQR), g/d	85.9 (68.1 to 107.1)
Daily saturated fat intake, median (IQR), g/d	20.7 (16.4 to 26.5)
Energy intake contributed from NOVA groups, mean (SD), %	
Minimally processed foods	37.5 (11.0)
Males	36.6 (11.1)
Females	38.2 (10.9)
Processed culinary ingredients	2.4 (3.1)
Males	2.3 (3.1)
Females	2.5 (1.1)
Processed foods	15.1 (5.3)
Males	15.1 (5.5)
Females	15.4 (5.2)
UPFs	45.0 (11.7)
Males	46.0 (11.8)
Females	43.9 (11.4)
Age at outcome assessment, median (IQR), y	5.0 (5.0 to 5.1)
Organized physical activity, median (IQR), h/wk	2.0 (1.0 to 3.0)
Body mass index *z* score, median (IQR)	0.3 (−0.3 to 0.9)
Waist to height ratio *z* score, mean (SD)	−0.3 (1.0)
Subscapular skinfold thickness *z* score, mean (SD)	0.2 (1.1)
Triceps skinfold thickness *z* score, mean (SD)	0.5 (1.1)
With obesity at 5 y of age (n = 2213)^c^	103 (4.7)
With obesity or overweight at 5 y of age, No. (%) (n = 2213)^c^	438 (19.8)
Males	257 (11.6)
Females	181 (8.2)

Abbreviation: UPF, ultraprocessed food.

^a^ Values in the family, birth, and childhood characteristics are the pooled values after multiple imputations (n = 10).

^b^ Questionnaire choices were “Caucasian White” and Black, East Asian, First Nations, Hispanic, Middle Eastern, mixed, other, South Asian, South-East Asian (grouped as other).

^c^ Four participants did not have obesity data but had other outcomes.

### UPF Intake

**Figure 1.**
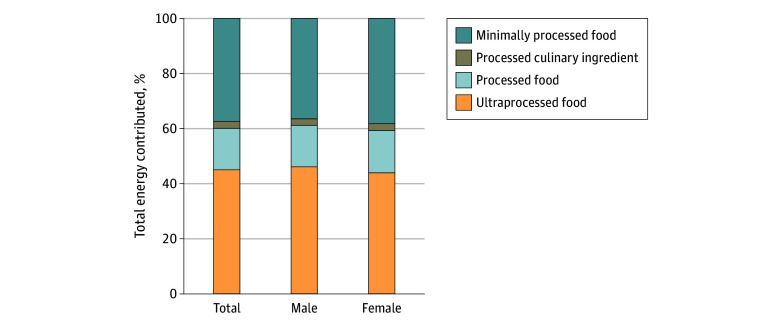
Energy Intake From Each NOVA Group at 3 Years of Age Among 2217 Participants in the CHILD Cohort Study Stacked bar graph shows total energy intake percentage contributed by each NOVA group in the total population and stratified by male and female participants.

On average, 45.0% of total daily energy intake came from UPF among children aged 3 years (Figure 1). Unprocessed and minimally processed foods contributed 37.5% of total daily energy intake, while processed culinary ingredients contributed 2.4% and processed food contributed 15.1% (Figure 1). Intake of UPF energy was higher among males than females (46.0% vs 43.9%; *P* < .001).

### Associations Between Preschool UPF Intake and School-Age Anthropometric Adiposity Indicators

**Figure 2.**
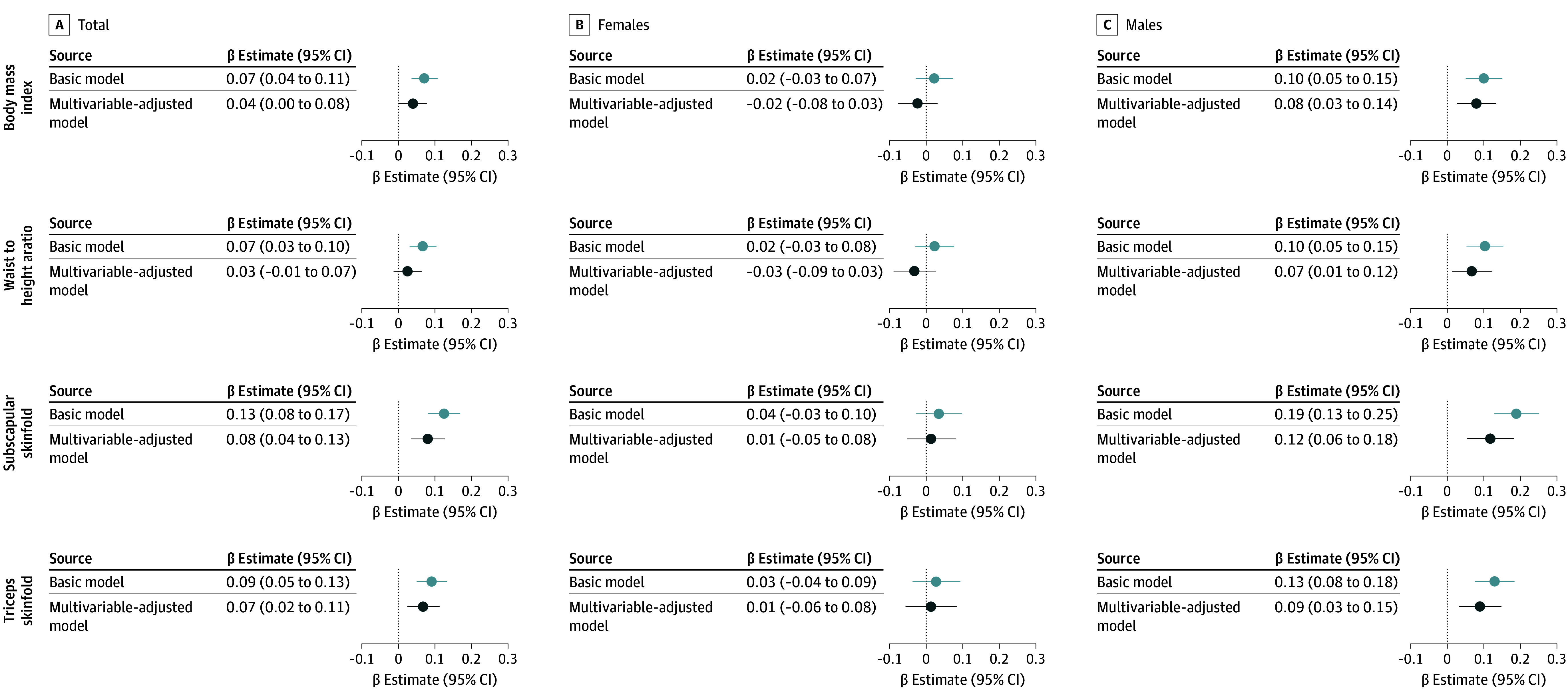
Ultaprocessed Food (UPF) Intake at 3 Years of Age and Anthropometric Adiposity Indicators at 5 Years Among 2217 Participants in the CHILD Cohort Study β Estimates with 95% CIs are calculated from linear regression analyses of the associations between every 10% increase in energy intake from UPF intake at 3 years of age and their associations with anthropometric adiposity indicators *z* scores (body mass index, waist to height ratio, and subscapular and triceps skinfold thickness) at 5 years of age. The basic model accounted for total energy intake. The multivariable-adjusted model accounted for the basic model plus maternal postsecondary degree, maternal UPF intake during pregnancy, household family income, child ethnicity, birth weight, exclusive breastfeeding at 6 months, older siblings, hours of organized physical activity, study site, and season of dietary assessment.

Among the total population, in the basic and multivariable-adjusted linear regression analysis, each 10% increase in UPF energy contribution was associated with higher BMI and subscapular and triceps skinfold thickness (Figure 2). Among males, in the multivariable-adjusted analyses, every 10% UPF intake increase was associated with higher *z* scores for BMI (β, 0.08; 95% CI, 0.03-0.14), waist to height ratio (β, 0.07; 95% CI, 0.01-0.12), subscapular skinfold thickness (β, 0.12; 95% CI, 0.06-0.18), and triceps skinfold thickness (β, 0.09; 95% CI, 0.03-0.15) (Figure 2). No significant associations were observed among females. These associations were consistent in the complete case analysis (eFigure 2 in Supplement 1). Among the participants with BMI measurements at baseline, we observed consistent results when additionally accounting for BMI at 3 years of age (eFigure 3 in Supplement 1). The patterns were similar among children with normal weight and children with overweight or obesity at baseline, although due to the smaller sample size, not all results retained statistical significance (eFigure 4 in Supplement 1).

To address the limitations of the NOVA classification, we derived highly processed foods using the UNC classification system reflective of North American diet and observed similar results (eFigure 5 in Supplement 1), highlighting the robustness of our NOVA UPF grouping. Furthermore, the associations of UPF and anthropometric adiposity indicators were also independent of sugar, sodium, and saturated fat intakes at 3 years of age (eFigure 5 in Supplement 1). These associations also remained when adjusting for the change in UPF intake between 3 and 5 years of age (eFigure 5 in Supplement 1). The observed associations persisted when examining UPF intake in grams (eFigure 6 in Supplement 1). Last, the site-stratified analyses showed similar patterns to the main results; however, not all the associations were retained across the 4 sites (eFigure 7 in Supplement 1).

### Associations Between Preschool UPF and School-Age Obesity Development

**Figure 3.**
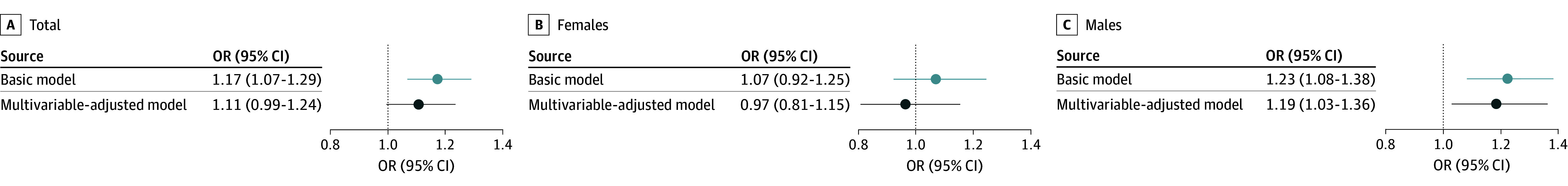
Ultraprocessed Food (UPF) Intake at 3 Years of Age and Overweight or Obesity Status at 5 Years of Age Among 2217 Participants in the CHILD Cohort Study Odds ratios (ORs) with 95% CIs are calculated from logistic regression analyses of the associations every 10% increase in energy intake from UPF intake at 3 years of age and their associations with overweight or obesity status at 5 years of age. The basic model accounted for total energy intake. The multivariable-adjusted model accounted for the basic model plus maternal postsecondary degree, maternal UPF intake during pregnancy, household family income, child ethnicity, birth weight, exclusive breastfeeding at 6 months, older siblings, hours of organized physical activity, study site, and season of dietary assessment.

Among all children, in the multivariable-adjusted logistic regression analyses, UPF intake was not associated with odds of overweight or obesity status. However, we observed that high UPF intake was associated with 1.19 (95% CI, 1.02-1.36) higher odds of living with overweight or obesity among males (Figure 3). There were no associations among females. Similar patterns were observed for obesity status; however, not all results remained statistically significant (eFigure 8 in Supplement 1).

## Discussion

Our cohort study is one of the largest longitudinal, population-based, Canadian studies examining the association of UPF with childhood obesity. We found that almost half of the daily energy intake among Canadian preschoolers came from UPF. Furthermore, we are the first to describe sex-driven associations between UPF intake and childhood adiposity and obesity in Canadian children.

### Comparison With Other Child UPF and Health Outcome Studies

Our findings on high UPF intake (45.0% of the total daily energy) in preschoolers are consistent with previous research. Among young children aged 2 to 5 years, the mean energy contribution from UPF was 48.0% according to the 2015 Canadian Community Health Survey data.^6^ Similarly, the Guelph Family Health Study found that UPF contributed toward 41.3% of total daily energy among children aged 1.5 to 5 years.^20^

Furthermore, we found a significant association between higher UPF intake at 3 years of age and higher *z* scores of anthropometric adiposity indicators at 5 years of age among Canadian children, with stronger effect estimates among males. The cross-sectional study from the Guelph Family Health Study did not report any associations between UPF intake and BMI *z* scores. However, comparison with our results is limited, as sex-stratified analysis was not performed,^20^ which might have been limited by the study’s sample size (N = 267). Similar to our findings, data from the Generation XXI birth cohort from Northern Portugal showed an association between high UPF consumption at 4 years of age and BMI *z* scores at 10 years of age.^18^ In the Pelotas-Brazil birth cohort, higher scores of UPF intake were associated with greater BMI-for-age *z* scores from 2 to 4 years of age.^16^ However, in a small (N = 307) longitudinal study from São Leopoldo, Brazil, UPF intake at 4 years of age was not significantly associated with the change in BMI at 8 years of age, but it was associated with waist circumference.^15^ Possible reasons for these inconsistencies include the different populations studied and the lack of sex-stratified analyses. Therefore, additional studies are needed to assess the impact of UPF later in childhood.

### Potential Mechanisms in the Development of Obesity From UPF Intake

Although the underlying mechanism in obesity development from UPF intake is not well understood, several potential mechanisms have been proposed. UPF is characterized as energy dense,^3^ and excess energy intake is known to be associated with obesity development.^36^ Additionally, the high saturated fat, sodium, and sugar content make UPFs hyperpalatable, which may drive overconsumption. In our study, we accounted for total energy intake and in a sensitivity analysis, we further adjusted for these nutrients of concern. Despite these adjustments, the associations remained significant. While studies in children are lacking, adult studies have related emulsifiers and additives found in UPF to altered gut microbiome,^37,38^ also reflecting different associations in males vs females,^39^ which aligns with our findings.

Finally, previous studies have shown sex differences in diet intake since early childhood. For example, males are reported to consume more calorie-dense foods.^10^ This aligns with our study findings where UPF energy contribution was higher in males. Additionally, sex differences are well-known in obesity and regional fat distributions. Studies documented that males tend to store fat in the abdominal regions, including both subcutaneous and visceral fat, while females tend to store in the gluteofemoral regions.^40^ Future studies should consider total and regional body composition, including lean, fat, or visceral fat mass.

### Study Importance and Implications

Given that Canadian children’s dietary patterns have shifted toward high UPF intake^6^ and that obesity tracks throughout life,^11^ our results may hold public health and clinical implications. Our study shows that UPF is associated with higher BMI, waist to height ratio, and subscapular and triceps skinfold thicknesses, independently of energy, sugar, saturated fat, and sodium intake, the change in diet (eg, UPF intake from 3 to 5 years of age), and baseline BMI. Although our effect estimates are small, they reflect a *z* score increase from every 10% UPF energy contribution and may be significant on a population level. Promoting healthy food choices in early childhood can lead to better dietary patterns and improved long-term nutrient intake.^41^ Our results can support existing public health policies such as front-of-package-labeling regulation or develop new policies.^42^ Understanding the role UPF plays in childhood obesity can allow health care professionals to provide tailored dietary recommendations for obesity prevention. This work can also inform future studies, such as UPF subgroup studies (currently the NOVA system considers plant-based alternatives as UPF), studies examining other characteristics of UPF including the use of emulsifiers,^5^ potential physiological mediators in the development of childhood obesity, and mechanistic studies.

### Strengths and Limitations

To the best of our knowledge, this is one of the first prospective Canadian studies to examine early childhood UPF intake and obesity development and one of the first worldwide to demonstrate associations primarily driven by males. The large and detailed CHILD database enabled our study to adjust for various potential confounders, including maternal UPF intake. In addition to BMI *z* scores, we also calculated *z* scores of waist to height ratio and skinfold thicknesses. Waist to height ratio may be a better indicator of central obesity, while skinfold thickness are shown to estimate adult adiposity more accurately than BMI.^43^ Using both measures alongside BMI is useful for assessing childhood obesity.^44,45,46^

Our study also holds some limitations. Although we used a detailed 112-item FFQ, this tool is not ideal for capturing UPF intake. Furthermore, despite being the most widely used, the NOVA system is limited by the criteria interpretability.^47^ To deal with this, 2 researchers independently mapped the FFQ items using the NOVA system and followed the commonly reported conservative approach to classify ambiguous items. In addition, we replicated the results using the UNC system, which is adapted for the North American diet, and observed similar results. To assess the potential selection bias due to loss to follow-up, we conducted 2 nonresponse analyses and found that participants lost to follow-up shared similar characteristics to the participants included in the main analyses. We ran multiple imputations for confounders in our multivariable-adjusted model to reduce bias due to missing covariate data. Additionally, the complete case analyses showed similar results, with slightly smaller effect estimates compared with the analyses on imputed data. Last, despite adjusting for several potential confounders, due to the observational design of our study, residual confounding (eg, family eating behaviors) might be possible.

## Conclusions

In this cohort study of Canadian children, UPF intake represented almost half of all energy intake, which warrants public health attention. Our results also suggest sex-driven associations between preschool UPF intake and obesity development. Future studies should examine the underlying mechanism and determine the long-term impact of UPF on youth health.

## Notes

Supplement 1. **eFigure 1.** Flowchart of the Study Participants Included in the Analysis **eTable 1.** Descriptive Characteristics of the Original and Multiple Imputed Datasets in the CHILD Cohort Study **eTable 2.** Descriptive Characteristics of Participants Enrolled in the Study at Birth (N = 3454) and Non-Response Analyses **eFigure 2.** Associations of Ultraprocessed Food Intake and Anthropometric Adiposity Indicator Z Scores Among Participants With No Missing Covariate Data (Complete Case Analysis) in the CHILD Cohort Study (n = 1617) **eFigure 3.** Associations of Ultraprocessed Food Intake and Anthropometric Adiposity Indicator Z Scores Additionally Accounting for Baseline Body Mass Index in the CHILD Cohort Study (n = 2145) **eFigure 4.** Associations of Ultraprocessed Food Intake and Anthropometric Adiposity Indicator Z Scores Stratified by Baseline Overweight or Obesity Status in the CHILD Cohort Study (n = 2138) **eFigure 5.** Associations of Ultraprocessed Food Intake and Anthropometric Adiposity Indicator Z Scores in the CHILD Cohort Study (Sensitivity Analyses) (n = 2217) **eFigure 6.** Associations of Ultraprocessed Food Intake in Grams and Anthropometric Adiposity Indicator Z Scores in the CHILD Cohort Study (n = 2217) **eFigure 7.** Associations of Ultraprocessed Food Intake and Anthropometric Adiposity Indicator Z Scores Stratified by Study Sites in the CHILD Cohort Study (n = 2217) **eFigure 8.** Associations of Ultraprocessed Food Intake at 3 Years of Age With Obesity Status at 5 Years of Age (n = 2217) in the CHILD Cohort Study

Supplement 2.
Data Sharing Statement
