# Supplementary material for: Ultraprocessed Food Consumption and Obesity Development in Canadian Children
Source: JAMA Netw Open. 2025 Jan 31;8(1):e2457341. doi: 10.1001/jamanetworkopen.2024.57341 (PMC11786234; doi:10.1001/jamanetworkopen.2024.57341)
Supplement: Supplement 1. — eFigure 1. Flowchart of the Study Participants Included in the Analysis eTable 1. Descriptive Characteristics of the Original and Multiple Imputed Datasets in the CHILD Cohort Study eTable 2. Descriptive Characteristics of Participants Enrolled in the Study at Birth (N = 3454) and Non-Response Analyses eFigure 2. Associations of Ultraprocessed Food Intake and Anthropometric Adiposity Indicator Z Scores Among Participants With No Missing Covariate Data (Complete Case Analysis) in the CHILD Cohort Study (n = 1617) eFigure 3. Associations of Ultraprocessed Food Intake and Anthropometric Adiposity Indicator Z Scores Additionally Accounting for Baseline Body Mass Index in the CHILD Cohort Study (n = 2145) eFigure 4. Associations of Ultraprocessed Food Intake and Anthropometric Adiposity Indicator Z Scores Stratified by Baseline Overweight or Obesity Status in the CHILD Cohort Study (n = 2138) eFigure 5. Associations of Ultraprocessed Food Intake and Anthropometric Adiposity Indicator Z Scores in the CHILD Cohort Study (Sensitivity Analyses) (n = 2217) eFigure 6. Associations of Ultraprocessed Food Intake in Grams and Anthropometric Adiposity Indicator Z Scores in the CHILD Cohort Study (n = 2217) eFigure 7. Associations of Ultraprocessed Food Intake and Anthropometric Adiposity Indicator Z Scores Stratified by Study Sites in the CHILD Cohort Study (n = 2217) eFigure 8. Associations of Ultraprocessed Food Intake at 3 Years of Age With Obesity Status at 5 Years of Age (n = 2217) in the CHILD Cohort Study [file jamanetwopen-e2457341-s001.pdf]

Supplementary Online Content

Chen ZH, Mousavi S, Mandhane PJ, et al. Ultraprocessed food consumption and obesity development in Canadian children. *JAMA Netw Open*. 2025;8(1):e2457341. doi:10.1001/jamanetworkopen.2024.57341

eFigure 1. Flowchart of the Study Participants Included in the Analysis

eTable 1. Descriptive Characteristics of the Original and Multiple Imputed Datasets in the CHILD Cohort Study

eTable 2. Descriptive Characteristics of Participants Enrolled in the Study at Birth (N = 3454) and Non-response Analyses

eFigure 2. Associations of Ultraprocessed Food Intake and Anthropometric Adiposity Indicator *Z* Scores Among Participants with No Missing Covariate Data (Complete Case Analysis) in the CHILD Cohort Study (n = 1617)

eFigure 3. Associations of Ultraprocessed Food Intake and Anthropometric Adiposity Indicator Z Scores Additionally Accounting for Baseline Body Mass Index in the CHILD Cohort Study (n = 2145)

eFigure 4. Associations of Ultraprocessed Food Intake and Anthropometric Adiposity Indicator Z Scores Stratified by Baseline Overweight or Obesity Status in the CHILD Cohort Study (n = 2138)

eFigure 5. Associations of Ultraprocessed Food Intake and Anthropometric Adiposity Indicator Z Scores in the CHILD Cohort Study (Sensitivity Analyses) (n = 2217)

eFigure 6. Associations of Ultraprocessed Food Intake in Grams and Anthropometric Adiposity Indicator Z Scores in the CHILD Cohort Study (n = 2217)

eFigure 7. Associations of Ultraprocessed Food Intake and Anthropometric Adiposity Indicator Z Scores Stratified by Study Sites in the CHILD Cohort Study (n = 2217)

eFigure 8. Associations of Ultraprocessed Food Intake at 3 Years of Age With Obesity Status at 5 Years of Age (n = 2217) in the CHILD Cohort Study

This supplementary material has been provided by the authors to give readers additional information about their work.

© 2025 Chen ZH et al. *JAMA Network Open*.

eFigure 1. Flowchart of the Study Participants Included in the Analysis

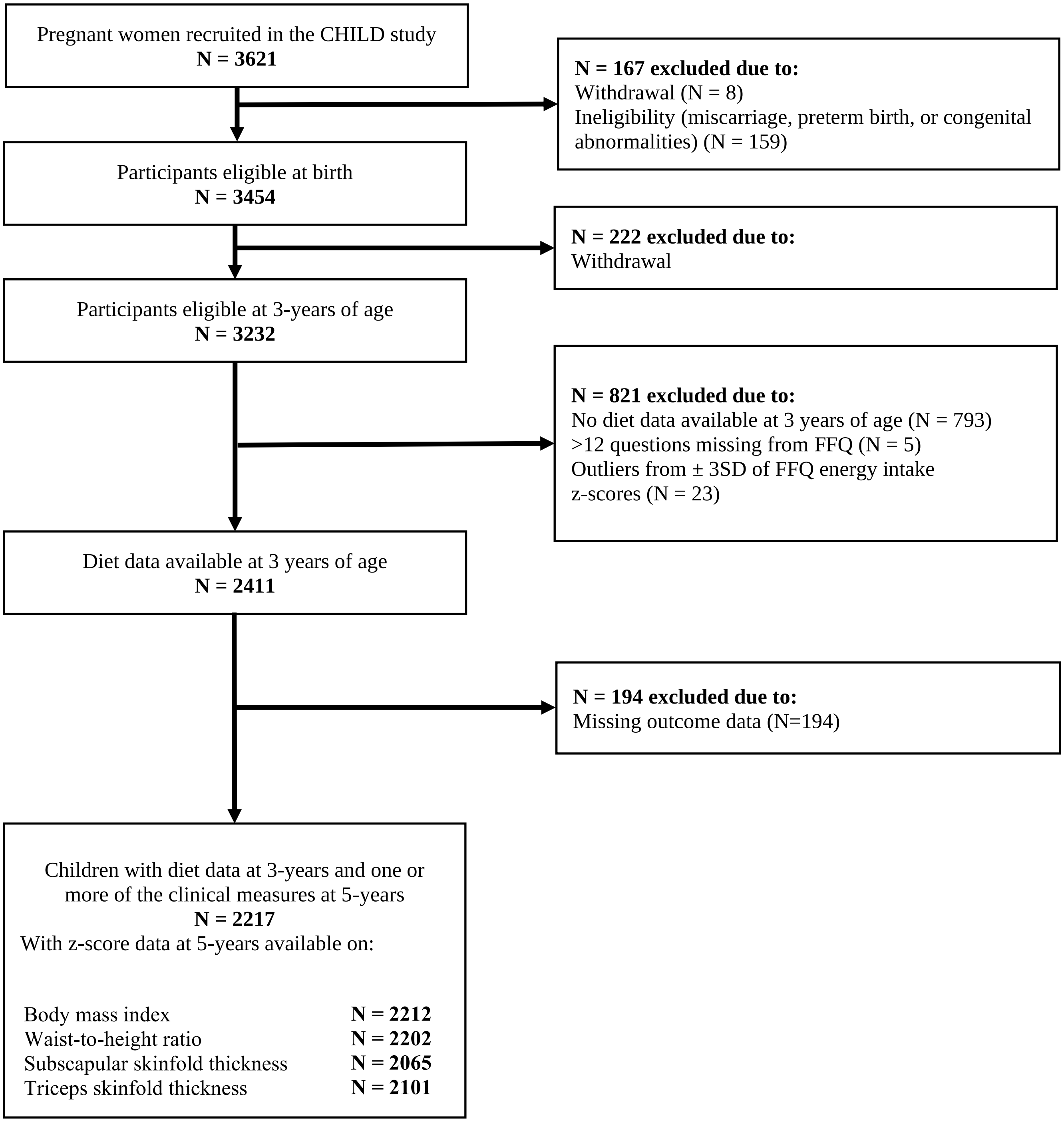

FFQ = Food frequency questionnaire; SD = standard deviation

© 2025 Chen ZH et al. *JAMA Network Open*.

eTable 1. Descriptive Characteristics of the Original and Multiple Imputed Datasets in the CHILD Cohort Study

	**Original (N = 2217)**	**Imputed (N = 2217)**
**Family Characteristics**		
Maternal Education (Post-Secondary Education vs. None)	1714 (79.1)	1742 (78.6)
Missing	50 (2.3)	0 (0.0)
Maternal Energy Intake Contributed from Ultraprocessed Food (%)	46.6 (10.5)	46.5 (10.5)
Missing	141 (6.4)	0 (0.0)
Annual Family Income		
< $100,000	778 (38.2)	892 (40.2)
≥ $100,000	1118 (54.9)	1178 (53.1)
Prefer Not to Say	139 (6.8)	147 (6.6)
Missing (n, %)	182 (8.2)	0 (0.0)
Other Siblings (Yes vs. No)	1014 (45.7)	1014 (45.7)
Missing	0 (0.0)	0 (0.0)
Study Site		
Edmonton	492 (22.2)	492 (22.2)
Manitoba	705 (31.8)	705 (31.8)
Toronto	483 (21.8)	483 (21.8)
Vancouver	537 (24.2)	537 (24.2)
Missing	0 (0.0)	0 (0.0)
**Birth Characteristics**		
Child Sex (Males vs. Females)	1175 (53.0)	1175 (53.0)
Missing	0 (0.0)	0 (0.0)
Birth Weight (kg)	3.5 (0.5)	3.5 (0.5)
Missing	44 (2.0)	0 (0.0)
Child Ethnicity (Caucasian vs. Not Caucasian)	1443 (65.3)	1447 (65.3)
Missing	8 (0.4)	0 (0.0)
Exclusive Breastfeeding at 6 Months (Yes vs. No)	402 (18.1)	414 (18.7)
Missing	33 (1.5)	0 (0.0)
**Childhood Characteristics**		
Season of Dietary Assessment		
Spring	603 (27.2)	603 (27.2)
Summer	563 (25.4)	563 (25.4)
Autumn	519 (23.4)	519 (23.4)
Winter	532 (24.0)	532 (24.0)
Missing	0 (0.0)	0 (0.0)

© 2025 Chen ZH et al. *JAMA Network Open*.

	Original (N = 2217)	Imputed (N = 2217)
**Childhood Characteristics**		
Daily Caloric Intake at Age Three (kcal/day)	1517.8 [1239.2, 1858.1]	1517.8 [1239.2, 1858.1]
Missing	0 (0.0)	0 (0.0)
Daily Sugar Intake at Age Three (g/day)	85.9 [68.1, 107.1]	85.9 [68.1, 107.1]
Missing	0 (0.0)	0 (0.0)
Daily Sodium Intake at Age Three (mg/day)	2168.3 [1730.0, 2780.5]	2168.3 [1730.0, 2780.5]
Missing	0 (0.0)	0 (0.0)
Daily Total Saturated Fat Intake at Age Three (g/day)	20.7 [16.4, 26.5]	20.7 [16.4, 26.5]
Missing	0 (0.0)	0 (0.0)
Energy Contributed from NOVA Groups at Age Three		
Minimally Processed Foods	37.5 (11.0)	37.5 (11.0)
Missing	0 (0.0)	0 (0.0)
Processed Culinary Ingredients	2.4 (3.1)	2.4 (3.1)
Missing	1 (0.1)	1 (0.1)
Processed Foods	15.1 (5.3)	15.1 (5.3)
Missing	0 (0.0)	0 (0.0)
Ultraprocessed Foods	45.0 (11.7)	45.0 (11.7)
Missing	0 (0.0)	0 (0.0)
Age at Outcome Assessment (years)	5.0 [5.0, 5.1]	5.0 [5.0, 5.1]
Missing	0 (0.0)	0 (0.0)
Organized Physical Activity at Age Five (hours/week)	2.0 [1.0, 3.0]	2.0 [1.0, 3.0]
Missing	303 (13.7)	0 (0.0)
Body Mass Index z-score at Age Five	0.3 [-0.3, 0.9]	0.3 [-0.3, 0.9]
Missing	4 (0.2)	4 (0.2)
Waist-to-Height Ratio z-score at Age Five	-0.3 (1.0)	-0.3 (1.0)
Missing	15 (0.7)	15 (0.7)
Subscapular Skinfold Thickness z-score at Age Five	0.2 (1.1)	0.2 (1.1)
Missing	152 (6.9)	152 (6.9)
Triceps Skinfold Thickness z-score at Age Five	0.5 (1.1)	0.5 (1.1)
Missing	116 (5.2)	116 (5.2)
With Obesity or Overweight at Age Five (Yes vs. No)	438 (19.8)	438 (19.8)
Missing	4 (0.2)	4 (0.2)
With Obesity at Age Five (Yes vs. No)	103 (4.7)	103 (4.7)
Missing	4 (0.2)	4 (0.2)

Values are frequency counts and percentages (%) for categorical variables, means and standard deviation (SD) for continuous variables with a normal distribution, or medians and interquartile range (IQR – 25th and 75th percentiles) for continuous variables with a skewed distribution.

© 2025 Chen ZH et al. *JAMA Network Open*.

eTable 2. Descriptive Characteristics of Participants Enrolled in the Study at Birth (N = 3454) and Non-response Analyses

	Participants enrolled in the study at birth (N = 3454)	Participants included in the analysis (n = 2217)	Participants without dietary and outcome data (n = 1237) [a]	Participants excluded from study due to no outcome data at age five years (n = 194) [b]
**Family Characteristics**				
Maternal Education (Post-Secondary Education vs. None)	2393 (76.4)	1714 (79.1)	679 (70.4) †	144 (76.6)
Maternal Energy Contribution from Ultraprocessed Food (%)	46.7 (10.8)	46.6 (10.5)	46.8 (11.3)	46.8 (11.2)
Annual Family Income				
< $100,000	857 (38.5)	778 (38.2)	79 (41.3)	56 (37.3)
≥ $100,000	1214 (54.5)	1118 (54.9)	96 (50.3)	83 (55.3)
Prefer Not to Say	155 (7.0)	139 (6.8)	16 (8.4)	11 (7.3)
Other Siblings (Yes vs. No)	1512 (46.4)	1014 (45.7)	498 (47.7)	84 (43.3)
Study Site				
Edmonton	735 (23.0)	492 (22.2)	243 (24.9) †	62 (32.0) ‡
Manitoba	988 (31.0)	705 (31.8)	283 (29.0) †	28 (14.4) ‡
Toronto	756 (23.7)	483 (21.8)	273 (28.0) †	59 (30.4) ‡
Vancouver	713 (22.3)	537 (24.2)	176 (18.1) †	45 (23.2) ‡
**Birth Characteristics**				
Child Sex (Males vs. Females)	1816 (52.6)	1175 (53.0)	641 (51.8)	89 (45.9)
Birth Weight (kg)	3.4 (0.5)	3.5 (0.5)	3.4 (0.5)	3.4 (0.5)
Child Ethnicity (Caucasian vs. Not Caucasian)	2162 (64.4)	1443 (65.3)	719 (62.7)	126 (65.3)
Exclusive Breastfeeding at 6 Months (Yes vs. No)	552 (18.1)	402 (18.4)	150 (17.5)	32 (17.2)

© 2025 Chen ZH et al. *JAMA Network Open*.

	Participants enrolled in the study at birth (N = 3454)	Participants included in the analysis (n = 2217)	Participants without dietary and outcome data (n = 1237) [a]	Participants excluded from study due to no outcome data at age five years (n = 194) [b]
**Childhood Characteristics**				
Age at Dietary Assessment (years)	3.1 [3.0, 3.1]	3.0 [3.0, 3.1]		3.2 [3.0, 3.2] ‡
Season of Dietary Assessment				
Spring	657 (27.0)	603 (27.2)		51 (26.3)
Summer	621 (25.5)	563 (25.4)		52 (26.8)
Autumn	578 (23.8)	519 (23.4)		51 (26.3)
Winter	577 (23.7)	532 (23.9)		40 (20.6)
Daily Caloric Intake (kcal/day) at Age Three	1639.8 [1239.3, 1871.5]	1517.8 [1239.2, 1858.1]		1550.4 [1238.2, 1885.3]
Energy Contributed from NOVA Groups at Age Three				
Minimally Processed Foods	37.5 (11.0)	37.5 (11.0)		36.9 (10.0)
Processed Culinary Ingredients	2.4 (3.1)	2.4 (3.1)		2.9 (3.2)
Processed Foods	15.1 (5.3)	15.1 (5.3)		14.3 (5.1)
Ultraprocessed Foods	45.0 (11.7)	45.0 (11.7)		44.6 (9.9)
With Overweight or Obesity at Age Three (Yes vs. No)	202 (7.1)	146 (6.8)		8 (5.7)
Age at Outcome Assessment (years)	5.1 [5.0, 5.1]	5.0 [5.0, 5.1]		5.0 [4.8, 5.0]
Organized Physical Activity at Age Five (hours/week)	2.0 [1.0, 3.0]	2.0 [1.0, 3.0]		2.0 [0.0, 3.0]

Values are frequency counts and percentages (%) for categorical variables, means and standard deviation (SD) for continuous variables with a normal distribution, or medians and interquartile range (IQR – 25th and 75th percentiles) for continuous variables with a skewed distribution based on non-imputed data.

[a] Non-responsive analysis was done between participants included in the analysis (n=2217) and participants lost to follow-up since enrollment (n=1237) using an independent sample t-test for normally distributed variables, a Mann–Whitney U test for non-normally distributed variables, and chi-square test for categorical variables. † p-value < 0.05.

[b] Non-responsive analysis was done between participants included in the analysis (n=2217) and participants excluded due to having no outcome data but have diet data (n=194) using an independent sample t-test for normally distributed variables, a Mann–Whitney U test for non-normally distributed variables, and chi-square test for categorical variables. ‡ p-value < 0.05.

© 2025 Chen ZH et al. *JAMA Network Open*.

eFigure 2. Associations of Ultraprocessed Food Intake and Anthropometric Adiposity Indicator *Z* Scores Among Participants with No Missing Covariate Data (Complete Case Analysis) in the CHILD Cohort Study (n = 1617)

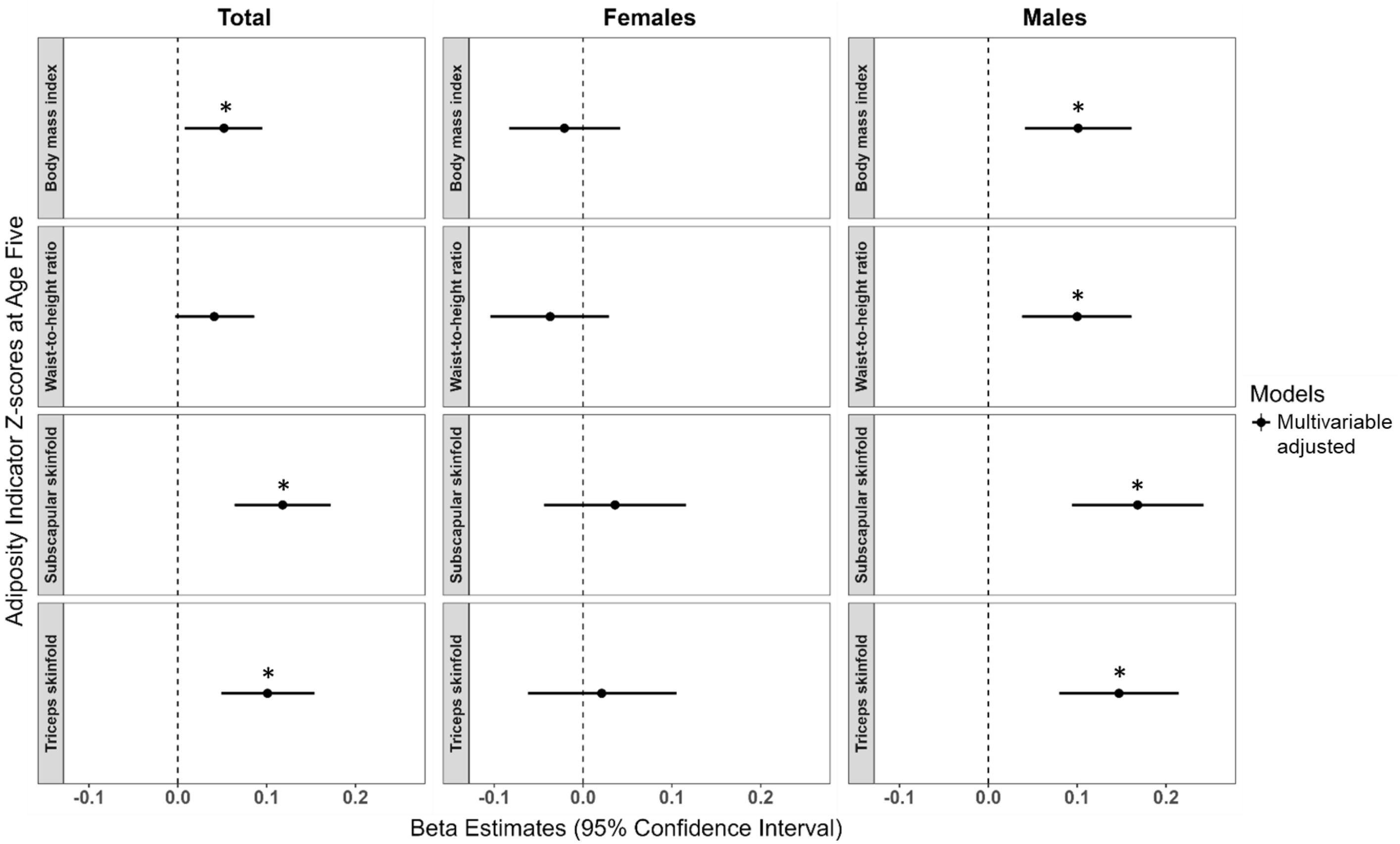

Values are beta estimates with 95% confidence intervals from linear regression analyses of every 10% increase in energy intake from ultraprocessed food (UPF) intake at age three years and their associations with anthropometric adiposity indicator z-scores (body mass index, waist-to-height ratio, subscapular and triceps skinfold thickness) at age five years, in the CHILD Cohort Study (N=1617). The multivariable-adjusted model accounted for total energy intake, maternal post-secondary degree, maternal UPF intake during pregnancy, household family income, child ethnicity, birth weight, exclusive breastfeeding at six months, older siblings, hours of organized physical activity, study site, season of dietary assessment, and body mass index at age three years. *p-value<0.05.

© 2025 Chen ZH et al. *JAMA Network Open*.

eFigure 3. Associations of Ultraprocessed Food Intake and Anthropometric Adiposity Indicator Z Scores Additionally Accounting for Baseline Body Mass Index in the CHILD Cohort Study (n = 2145)

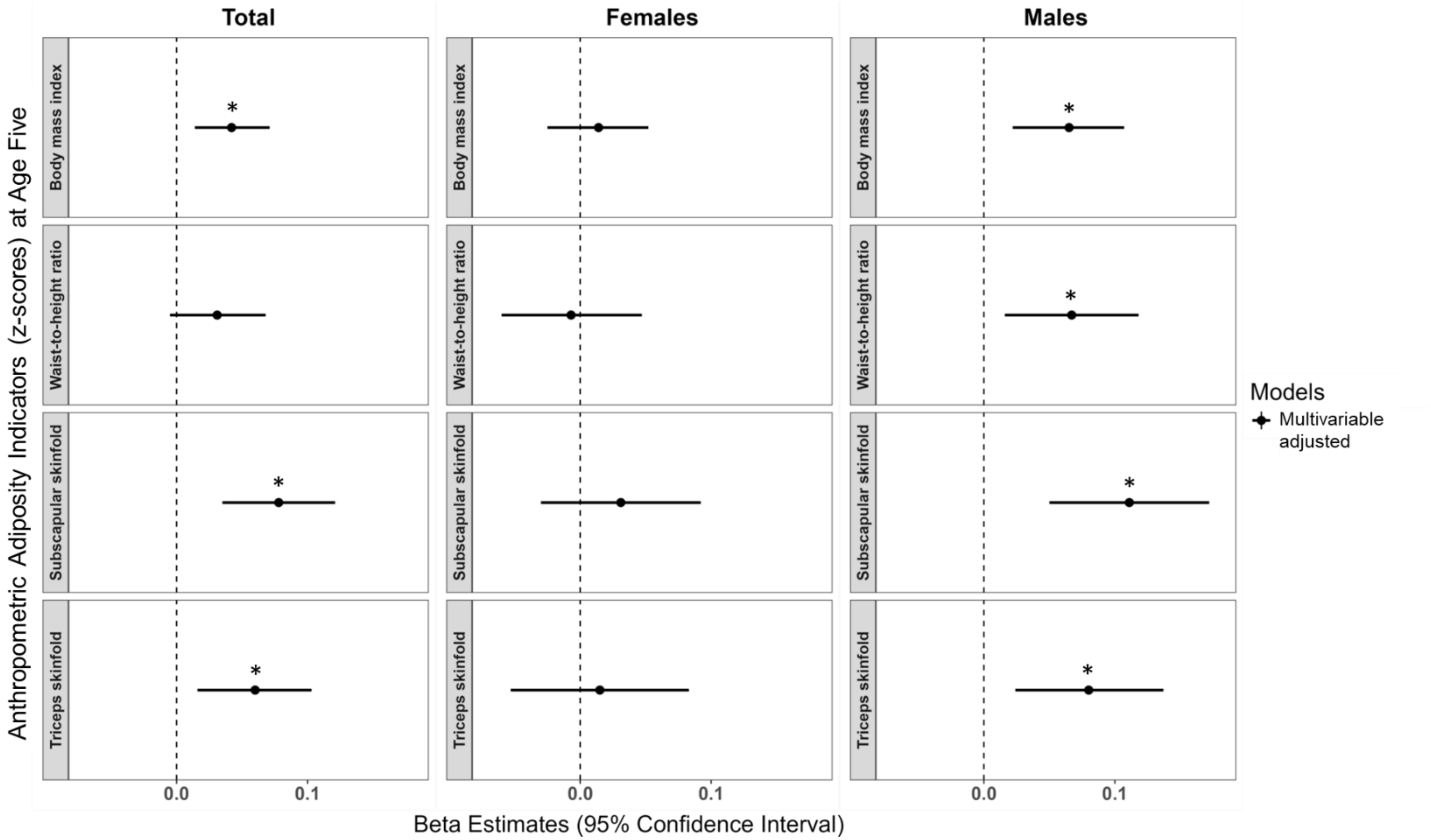

Values are beta estimates with 95% confidence intervals from linear regression analyses of every 10% increase in energy intake from ultraprocessed food (UPF) intake at age three years and their associations with anthropometric adiposity indicator z-scores (body mass index, waist-to-height ratio, subscapular and triceps skinfold thickness) at age five years, in the CHILD Cohort Study (N=2145). The multivariable-adjusted model accounted for total energy intake, maternal post-secondary degree, maternal UPF intake during pregnancy, household family income, child ethnicity, birth weight, exclusive breastfeeding at six months, older siblings, hours of organized physical activity, study site, season of dietary assessment, and body mass index at age three years. *p-value<0.05.

© 2025 Chen ZH et al. *JAMA Network Open*.

eFigure 4. Associations of Ultraprocessed Food Intake and Anthropometric Adiposity Indicator *Z* Scores Stratified by Baseline Overweight or Obesity Status in the CHILD Cohort Study (n = 2138)

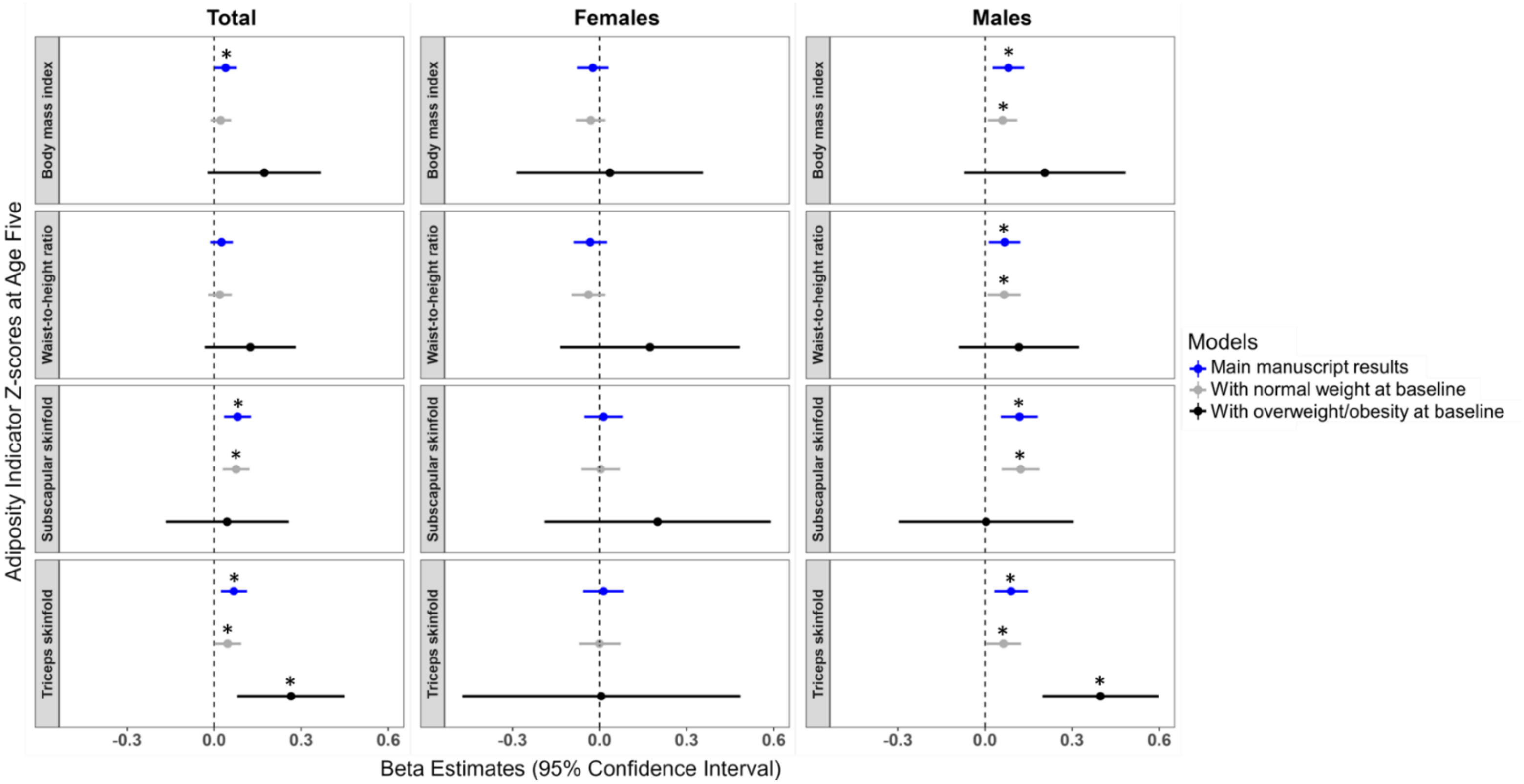

Values are beta estimates with 95% confidence intervals from linear regression analyses of every 10% increase in energy intake from ultraprocessed food (UPF) intake at age three years and their associations with anthropometric adiposity indicator z-scores (body mass index, waist-to-height ratio, subscapular and triceps skinfold thickness) at age five years, in the CHILD Cohort Study. Analyses are stratified among children with normal weight (n=1995) and those with overweight and obesity (n=143) at three years of age. The multivariable-adjusted model accounted for total energy intake, maternal post-secondary degree, maternal UPF intake during pregnancy, household family income, child ethnicity, birth weight, exclusive breastfeeding at six months, older siblings, hours of organized physical activity, study site, and season of dietary assessment. *p-value<0.05.

© 2025 Chen ZH et al. *JAMA Network Open*.

eFigure 5. Associations of Ultraprocessed Food Intake and Anthropometric Adiposity Indicator *Z* Scores in the CHILD Cohort Study (Sensitivity Analyses) (n = 2217)

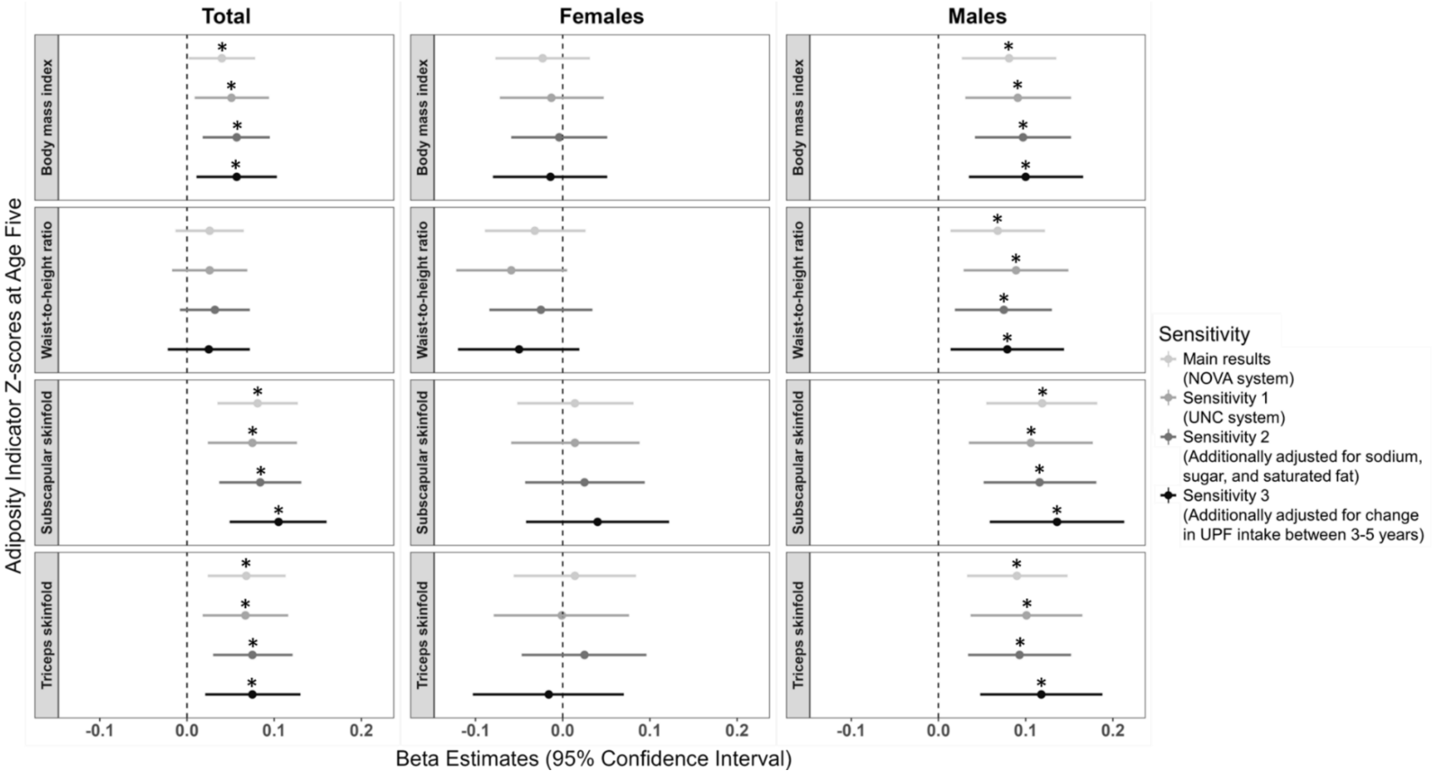

Values are beta estimates with 95% confidence intervals from linear regression analyses of every 10% increase in energy intake from ultraprocessed food (UPF) intake at age three years and their associations with anthropometric adiposity indicator z-scores at age five years, in the CHILD Cohort Study (N=2217). Main results = associations reported in the manuscript; Sensitivity 1 = highly processed food according to the classification system developed by the University of North Carolina (UNC) that is reflective of a North American diet; Sensitivity 2 = additional adjustment for total sugar, sodium, and saturated fat intake in the multivariable-adjusted model; Sensitivity 3 = additional adjustment for change in UPF intake between three and five years of age. The multivariable-adjusted model accounted for total energy intake, maternal post-secondary degree, maternal UPF intake during pregnancy, household family income, child ethnicity, birth weight, exclusive breastfeeding at six months, older siblings, hours of organized physical activity, study site, and season of dietary assessment. *p-value<0.05.

© 2025 Chen ZH et al. *JAMA Network Open*.

eFigure 6. Associations of Ultraprocessed Food Intake in Grams and Anthropometric Adiposity Indicator *Z* Scores in the CHILD Cohort Study (n = 2217)

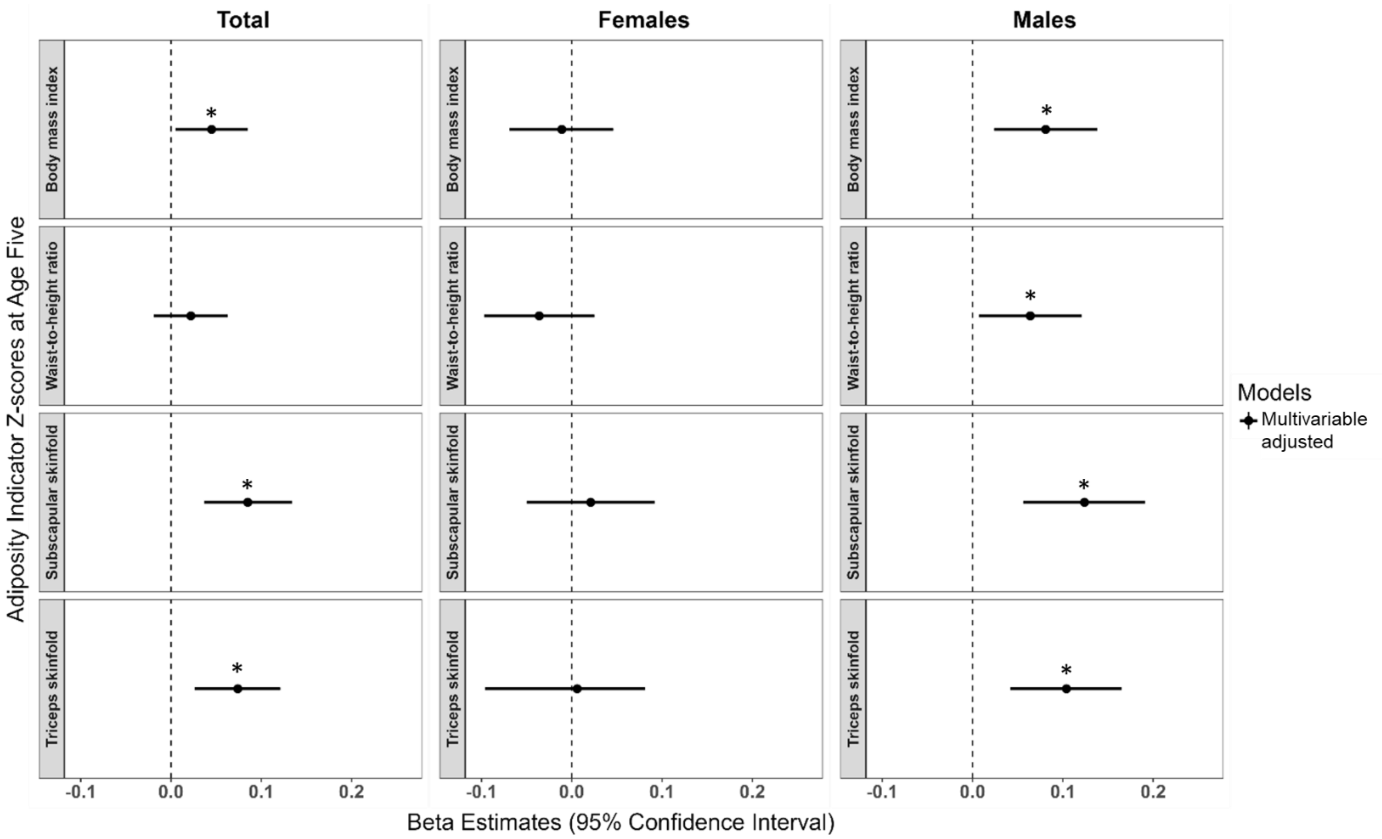

Values are beta estimates with 95% confidence intervals from linear regression analyses of every 100 grams of ultraprocessed food (UPF) intake at age three years and their associations with anthropometric adiposity indicator z-scores (body mass index, waist-to-height ratio, subscapular and triceps skinfold thickness) at age five years, in the CHILD Cohort Study (N=2217). The multivariable-adjusted model accounted for total energy intake, maternal post-secondary degree, maternal UPF intake during pregnancy, household family income, child ethnicity, birth weight, exclusive breastfeeding at six months, older siblings, hours of organized physical activity, study site, and season of dietary assessment. *p-value<0.05.

© 2025 Chen ZH et al. *JAMA Network Open*.

eFigure 7. Associations of Ultraprocessed Food Intake and Anthropometric Adiposity Indicator *Z* Scores Stratified by Study Sites in the CHILD Cohort Study (n = 2217)

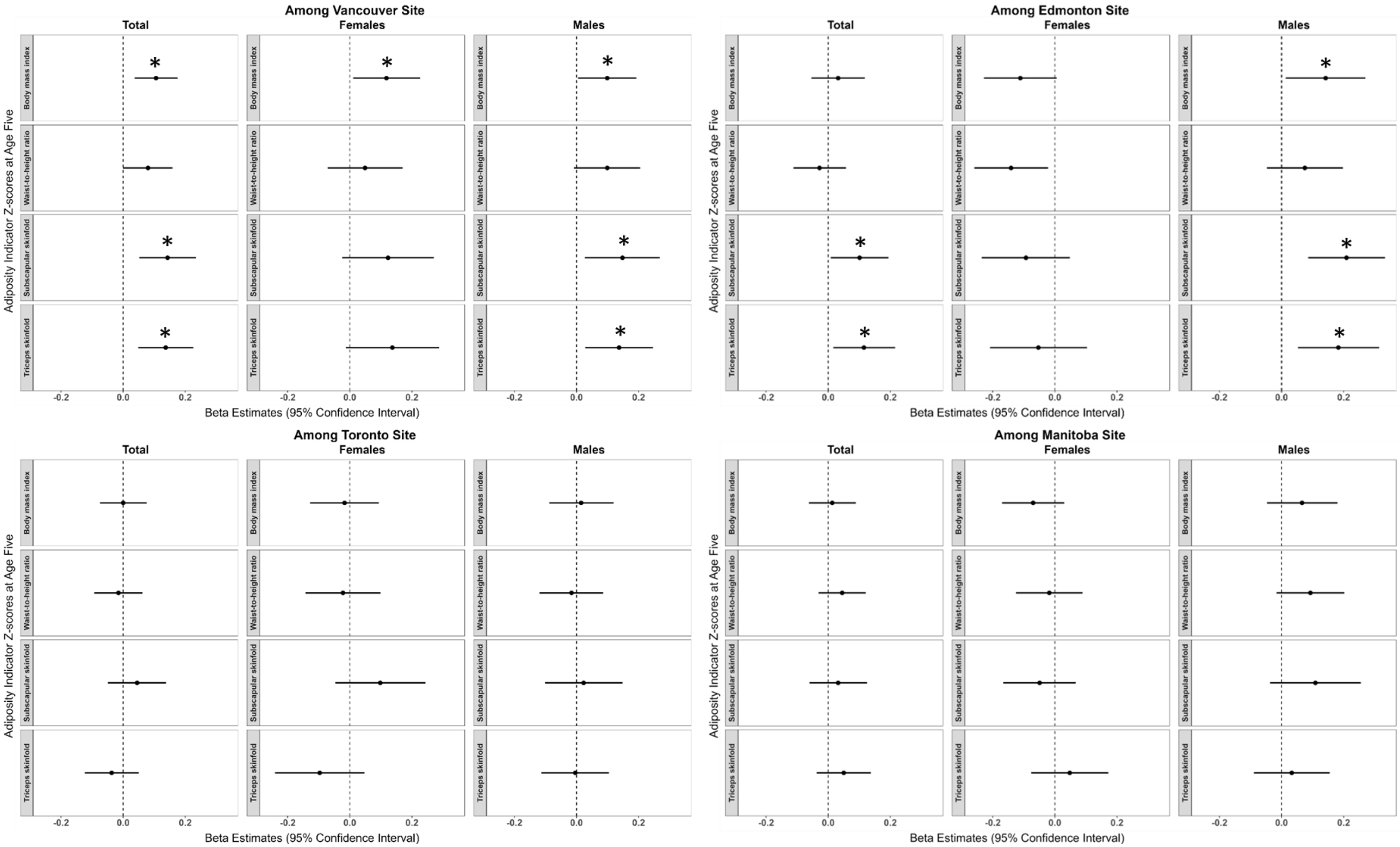

© 2025 Chen ZH et al. *JAMA Network Open*.

Values are beta estimates with 95% confidence intervals from linear regression analyses of every 10% increase in energy intake from ultraprocessed food (UPF) intake at age three years and their associations with anthropometric adiposity indicator z-scores (body mass index, waist-to-height ratio, subscapular and triceps skinfold thickness) at age five years, in the CHILD Cohort Study. Analyses are stratified by study site: Vancouver (N=537), Edmonton (N=492), Toronto (N=483), and Manitoba (N=705). The multivariable-adjusted model accounted for total energy intake, maternal post-secondary degree, maternal UPF intake during pregnancy, household family income, child ethnicity, birth weight, exclusive breastfeeding at six months, older siblings, hours of organized physical activity, and season of dietary assessment. *p-value<0.05.

© 2025 Chen ZH et al. *JAMA Network Open*.

eFigure 8. Associations of Ultraprocessed Food Intake at 3 Years of Age with Obesity Status at 5 Years of Age in the CHILD Cohort Study (n = 2217)

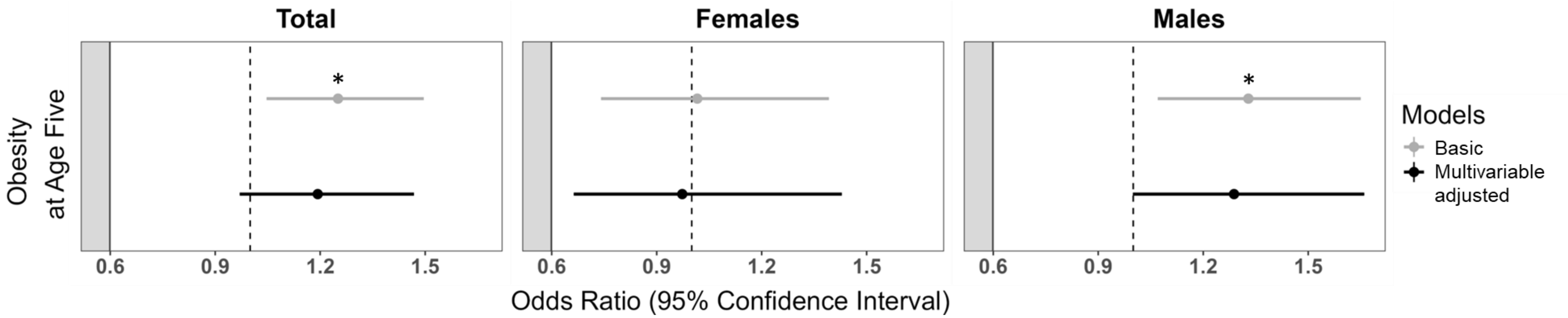

Values are odds ratio with 95% confidence intervals from logistic regression analyses of every 10% increase in energy intake from ultraprocessed food (UPF) intake at age three years and their associations with obesity status at age five years, in the CHILD Cohort Study (N=2217). The basic model accounted for total energy intake. The multivariable-adjusted model accounted for the basic model + maternal post-secondary degree, maternal UPF intake during pregnancy, household family income, child ethnicity, birth weight, exclusive breastfeeding at six months, older siblings, hours of organized physical activity, study site, and season of dietary assessment. *p-value<0.05

© 2025 Chen ZH et al. *JAMA Network Open*.
