# Supplementary material for: Ultraprocessed Food Consumption and Obesity Development in Canadian Children
Source: JAMA Netw Open. 2025 Jan 31;8(1):e2457341. doi: 10.1001/jamanetworkopen.2024.57341 (PMC11786234; doi:10.1001/jamanetworkopen.2024.57341)
Supplement: Supplement 2. — Data Sharing Statement [file jamanetwopen-e2457341-s002.pdf]

Data Sharing Statement

Chen. Ultraprocessed Food Consumption and Obesity Development in Canadian Children. *JAMA Netw Open*. Published January 31, 2025. doi:10.1001/jamanetworkopen.2024.57341

Data
Data available: Yes
Data types: Other (please specify)
Additional Information: Data described in the manuscript and analytic code will be made available upon request pending approval from CHILD's Access and Publication Committee and the CHILD Study National Coordinating Centre.
How to access data: A list of variables available in the CHILD Cohort Study is available at https://childstudy.ca/for-researchers/study-data/. More information about data access for the CHILD Cohort Study can be found at https://childstudy.ca/for-researchers/data-access/.
When available: With publication

Supporting Documents
Document types: None

Additional Information
Who can access the data: Data described in the manuscript and analytic code will be made available upon request pending approval from CHILD's Access and Publication Committee and the CHILD Study National Coordinating Centre.
Types of analyses: Researchers interested in collaborating on a project and accessing CHILD Cohort Study data should contact the Study's National Coordinating Centre (NCC) to discuss their needs before initiating a formal request. To contact the NCC, please email child@mcmaster.ca.
Mechanisms of data availability: Researchers interested in collaborating on a project with us (kozeta.miliku@utoronto.ca) and accessing CHILD Cohort Study data should contact the Study's National Coordinating Centre (NCC) to discuss their needs before initiating a formal request. To contact the NCC, please email child@mcmaster.ca.
